# Acoustic Emission Monitoring of Progressive Damage of Reinforced Concrete T-Beams under Four-Point Bending

**DOI:** 10.3390/ma15103486

**Published:** 2022-05-12

**Authors:** Deba Datta Mandal, Mourad Bentahar, Abderrahim El Mahi, Alexandre Brouste, Rachid El Guerjouma, Silvio Montresor, François-Baptiste Cartiaux

**Affiliations:** 1 Laboratoire d’Acoustique de l’Université du Mans (LAUM), UMR 6613, Institut d’Acoustique—Graduate School (IA-GS), CNRS, Le Mans Université, Avenue Olivier Messiaen, CEDEX 9, 72085 Le Mans, France; deba_datta.mandal@univ-lemans.fr (D.D.M.); abderrahim.elmahi@univ-lemans.fr (A.E.M.); rachid.elguerjouma@univ-lemans.fr (R.E.G.); silvio.montresor@univ-lemans.fr (S.M.); 2 Laboratoire Manceau de Mathématiques, Le Mans Université, Avenue Olivier Messiaen, CEDEX 9, 72085 Le Mans, France; alexandre.brouste@univ-lemans.fr; 3 Osmos Group, 37 Rue de la Perouse, 75116 Paris, France; cartiaux@osmos-group.com

**Keywords:** reinforced concrete beam, four-point bending, acoustic emission (AE), *b-value*, average frequency, *RA-value*, support-vectors machine, unsupervised machine learning

## Abstract

Acoustic Emission (AE) is revealed to be highly adapted to monitor materials and structures in materials research and for site monitoring. AE-features can be either analyzed by means of physical considerations (geophysics/seismology) or through their time/frequency waveform characteristics. However, the multitude of definitions related to the different parameters as well as the processing methods makes it necessary to develop a comparative analysis in the case of a heterogeneous material such as civil engineering concrete. This paper aimed to study the micro-cracking behavior of steel fiber-reinforced reinforced concrete T-beams subjected to mechanical tests. For this purpose, four-points bending tests, carried out at different displacement velocities, were performed in the presence of an acoustic emission sensors network. Besides, a comparison between the sensitivity to damage of three definitions corresponding to the *b-value* parameter was performed and completed by the evolution of the *RA-value* and average frequency (*AF*) as a function of loading time. This work also discussed the use of the support-vector machine (SVM) approach to define different damage zones in the load-displacement curve. This work shows the limits of this approach and proposes the use of an unsupervised learning approach to cluster AE data according to physical and time/frequency parameters. The paper ends with a conclusion on the advantages and limitations of the different methods and parameters used in connection with the micro/macro tensile and shear mechanisms involved in concrete cracking for the purpose of in situ monitoring of concrete structures.

## 1. Introduction

Reinforced concrete is predominantly used in civil engineering structures for its desirable mechanical properties. However, the decline in strength of such structures is very common and is generally attributed to increased service loading, ageing, fatigue, corrosion, environmental impacts, etc. [1]. The presence of damage and progressive deterioration of an existing concrete structure may result in poor performance, and even incur failure under service loading. Therefore, it is of paramount importance to understand the involved damage mechanisms and their symptoms for proper maintenance of these structures ensuring safety, enhanced durability, and economic operations.

Acoustic emission is revealed to be highly adapted to monitor materials and structures in different fields (e.g., civil engineering) in a nondestructive way [2,3,4]. The AE technique is based on the detection of the rapid release of energy in the form of transient elastic stress waves from a localized source within a material [5,6]. These transient AE signals are usually burst type and can be related to different types of sources such as initiation/propagation of crack fronts, yielding, failure of bonds, fiber failure, delamination, etc. [7,8]. Despite the lack of a standard procedure that can be used for all types of structures [9], AE remains widely accepted and used in structural health monitoring of civil engineering structures [1] thanks to its non-invasive character. The main advantage of the AE-based technique is its ability to perform real-time continuous monitoring of the entire volume of a structure [10]. Consequently, it can provide early information about incipient damage and thereby prevent catastrophic failure of a structure. Although acoustic emission-based studies were introduced long ago [9], AE-based assessment of materials and/or structures has gained significant traction in recent times. Various AE features can be determined from an AE-based experiment, where it becomes possible to link the AE signals with the involved types of damage [6]. AE event rate analysis was used to monitor the deterioration processes during creep and fatigue tests in association with failure prediction models [11,12]. AE event rate was also used to study salt crystallization in limestones [13]. Some research revealed that AE signals can also be used to understand the nonlinear relaxation (slow dynamics) of concrete taken at the initial and micro-cracked states [14,15].

AE features have also been analyzed by means of physical considerations in order to evaluate the main damage mechanisms responsible for the detected AE events. Features such as rise time, signal amplitude, and average frequency were studied in different research in order to access the involved fracture created in different brick and mortar types [16,17,18]. The severity of damage can be evaluated through the seismology parameter called the *b-value.* Being sensitive to the coalescence of micro-cracks into macro-cracks, it has been used in different studies in order to detect and monitor critical damage development [19,20,21,22]. Based on the shift of the *b-value* and AE parameters, a statistical and fractal analysis was performed by Carpinteri et al. in which they showed that the energy release during the microcracking happens in a fractal domain and where the *b-value* was found to be related to the fractal dimension [23,24,25].

A waveform-based analysis of AE signals requires broadband AE sensors and high sampling rates. In the case of heterogeneous materials, the involved elastic waves can be attenuated, which makes the study of high-frequency components difficult [26]. A simplified signal analysis method was then proposed in which one takes into account the rise time, amplitude, and average frequency of the AE signals [18,27,28]. However, the use of the proposed method depends on the distance between the source and the sensor, which should be compensated in some cases as in ref. [29].

Several works have used AE-based techniques to classify crack modes in reinforced concrete beams subjected to bending [17,30,31,32,33]. Ohno and Ohtsu [27] successfully used simplified Green’s functions for moment tensor analysis (SiGMA) to quantitatively classify cracks in terms of tensile (mode I) and shear (mode II) in concrete structures. Probabilistic methods, such as Gaussian mixture modeling (GMM), have also been used to classify cracks using AE data [1,34]. Very few studies were found on support vector machine (SVM)-based classification of AE data. Das et al. [35] classified cracking modes in steel fiber-reinforced concrete beam under bending and strain hardening cementitious composite samples under tension loading using Gaussian Mixture Models and a SVM-combined framework. The crack mode classification showed that more events were caused by matrix cracking during strain hardening, while during the softening phase, a larger number of events were found to be associated with fiber pull out. R. V. Sagar [36] classified the crack modes in reinforced concrete beams using GMM and the results were validated with the help of SVM.

The AE events were localized with the help of localization algorithms [30] by considering the arrival time of longitudinal AE waves across sensors, and it was observed that localization can be improved with the help of a well-arranged network of sensors. Mirgal et al. [37], with the help of their localization algorithm, found that sensors placed in a zigzag fashion can provide better localization of pencil lead break (PLB) sources than using a rectangular or circular arrangement on the surface of concrete slab. Soulioti et al. [31] studied the effect of fiber content on fracture modes in concrete beams using AE data. They observed that for unreinforced concrete, the dominating fracture mode is tensile in nature, whereas in the case of reinforced concrete the dominating mode of fracture changes to shear as fiber content increases. Anay et al. [38] monitored cement paste-based specimens by AE under compressive loading and classified progressive failure mechanisms. They identified three distinct crack behaviors, namely microcrack initiation, crack extension, and unstable crack growth.

The huge quantity of AE data makes the reduction of their dimensionality necessary. For that purpose, principal component analysis (PCA) was often used in different applications including concrete and composites [38,39]. PCA is often followed by a clustering using an unsupervised technique. In that sense, Sena et al. [40] studied the micro cracking of steel fiber-reinforced concrete beams under three-point bending. The multivariable clustering revealed the existence of two types of failures, namely matrix failure and steel fiber/matrix debonding. Calabrese et al. [41] applied PCA to reduce the dimension of AE data obtained from a four-point bending test on concrete beams. The Kohonen’s self-organizing map (SOM) algorithm, which is an artificial neural net based on an unsupervised learning method, was implemented with the aim to discern clusters and relate them to the AE patterns. Sun et al. [42] investigated the failure process involved in crumb rubber concrete under four-point bending using AE techniques. Various clustering methods, namely k-means, fuzzy c-means (FCM), self-organizing mapping (SOM), Gaussian mixture model (GMM), hierarchical model, and density peak clustering were used in order to find the best algorithm for that particular scenario, and the density peak algorithm was found to produce best results. The main advantage of the AE-based technique is its ability to monitor the entire volume of a structure in real time [10]. Hence, AE monitoring can provide early information about incipient damage and thereby prevent catastrophic failure of a structure.

The present research work focused on the characterization of civil engineering concrete T-beams reinforced with steel bars subjected to four-point bending quasi-static tests. Steel bars prevent fragility and associated catastrophic failure and improve the post-peak behavior. They consequently offer a considerable enhancement of the load-carrying capacity and delayed failure was obtained [31]. Mechanical tests were monitored with AE sensors in order to extract the different AE signals emitted during the creation and propagation of micro-cracks. In order to highlight the dependence of the AE activity on the loading rates, bending tests were performed at different rates and AE signals were analyzed according to different time and frequency domain parameters. According to the literature, three different definitions of *b-value* can be used. This work compared the three definitions of the of *b-value* parameter, used in geophysics to study the fracture process in rocks, and drew a conclusion about their relevance in the case of the damaged concrete samples. AE data analysis was then performed with the help of specific waveform parameters, namely *RA-value* (rise time to maximum amplitude) and AF (average frequency). The use of a supervised learning approach based on the support vector machine (SVM) allowed us to separate the load-displacement curve into different areas according to the involved damage mechanisms. Moreover, unsupervised machine learning for clustering AE data allowed us to identify three clusters which were revealed to be frequency independent. This work ends by presenting the time/frequency characteristics of the AE signals according to the involved damage mechanisms.

## 2. Theoretical Background

Various AE features can be collected during an experiment with the help of a sophisticated AE monitoring system. Upon analyzing the AE features by using suitable algorithms, a good insight about the damage stage can be ascertained. Some useful algorithms that are usually used in AE based analysis are presented in the following.

### 2.1. Average Frequency (AF) and RA Value (RA)

**Figure 1:**
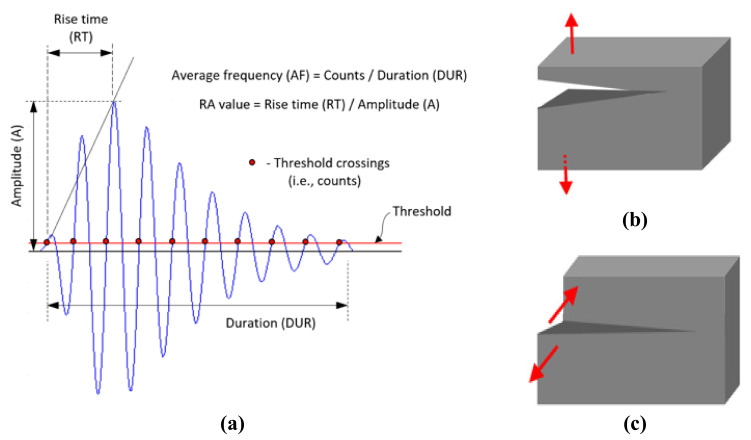
Schematic representation of (**a**) an AE signal and some important AE parameters, (**b**) tensile crack, and (**c**) shear crack.

Average frequency and *RA value* are two important quantities that can be used to determine crack modes in concrete structures [43]. Figure 1a presents the main parameters used to obtain the AF and RA of an AE waveform. Average Frequency (AF) is defined as the ratio between the number of counts and duration of an AE waveform. It basically determines the number of threshold crossings per unit time of an AE waveform.

(1) $$\mathrm{Average}\;\mathrm{Frequency}\;\left(\mathrm{AF}\right)\left[\mathrm{in}\;\mathrm{kHz}\right]=\frac{\mathrm{Counts}}{\mathrm{Duration}}$$

where “Counts” indicates the number of times the signal amplitude exceeds the fixed threshold over the entire duration of the AE waveform. *RA value* of an AE waveform is defined as the ratio between the rise time and amplitude:

(2) $$RA\;value\left[\mathrm{in}\mu s/V\right]=\frac{\mathrm{Rise}\;\mathrm{time}\;}{\mathrm{Amplitude}}$$

AE waveforms generated due to tensile cracks (Figure 1b) have shorter rise time [17,44]. Hence, tensile-type cracks usually generate AE signals with lower *RA values* and higher AF. On the other hand, in the case of shear-type cracks (Figure 1c), the AE waveforms are longer, *RA values* are relatively higher, and AF is lower [44].

### 2.2. The Three Different b-Values

The *b-value* analysis methods are generally used in geophysics to study the fracture process in rocks [45]. It uses amplitude of AE events. According to the literature, there are three different types of *b-value*, namely *b*_1_-*value*, *b*_2_-*value*, and *b*_3_-*value*.

#### 2.2.1. *b*_1_-*Value*

The pioneer work using the *b*_1_*-value* to assess the progressive failure was carried out by Shiotani et al. [46]. The authors suggested that the *b*_1_*-value* has the potential to be a precursor of slope failure. The expression of *b*_1_*-value* is given as:

(3) $$b_{1}=\frac{log_{10}N\left(\mu -\alpha_{1}\times \sigma \right)-log_{10}N\left(\mu +\alpha_{2}\times \sigma \right)}{(\alpha_{1}+\alpha_{2})\sigma }\;$$

where *N* is the number of recent events, *μ* is the mean value of the amplitudes of those recent events, *σ* is the standard deviation of the amplitudes of the same group of events, and *α*_1_ and *α*_2_ are empirical constants that specify which part of the population is considered. Usually, *α*_1_ and *α*_2_ are considered to be 0 and 1, respectively. 

Although *b*_1_*-value* was first used to study the fracture process in rocks, it was later used by many researchers to study fracture process in concrete [47,48]. Researchers in refs. [47,49] found that micro-cracks lead to relatively higher values of *b*_1_*-value* and macro-cracks lead to lower *b*_1_*-value*. Hence, a decrease in the *b*_1_*-value* may indicate successive accumulation of stress associated with a propagating rupture front [47].

#### 2.2.2. *b*_2_-*Value*

In the field of seismology, C. F. Richter and B. Gutenberg proposed an empirical relationship between earthquake frequency and magnitude, which is commonly known as the GR law, and the exponent of this law is known as the *b-value* [50]. The expression for the seismic *b-value* is given as:

(4) $$N\left(\geq M\right)=10^{a-b*M}\;\;\;\;\;or,\;\;\;\;log\left(N\right)=a-b*M\;$$

where *N* is the number of earthquakes whose magnitude is ≥M. a and  b are constants in an area over a specified span of time. [51,52]. Due to the resemblance between acoustic emission in materials and earthquakes, the *b-value* analysis has been incorporated in the progressive damage assessment of concrete structures. However, since the peak amplitude of an acoustic emission signal is measured in decibels (dB), and on the other hand, the magnitude of an earthquake is measured in the Richter Scale that considers the logarithm of seismic wave amplitude [45], it is necessary to use an adapted formula for the calculation of the “new” *b-value*, noted as the$\;b_{2}$-*value*:

(5) $$\log\left(N\right)=a_{2}-b_{2}*\left(\frac{A_{dB}}{20}\right)\;$$

*N* is the number of acoustic emission hits with an amplitude $\geq A_{dB}$, and $a_{2}$ is a constant that depends on the background noise (environment). The $b_{2}$-*value* is basically the slope of the log-linear graph of the frequency–magnitude distribution of AE hits.

#### 2.2.3. *b*_3_-*Value*

The *b*_3_-*value* was proposed by K. Aki [53]. He considered the discrete frequency distribution of earthquake magnitudes and, by means of statistical analysis, proposed the maximum likelihood of the *b-value* of the Gutenberg–Richter form of distribution. The expression of the *b*_3_-*value* is given as follows [45]:

(6) $$b_{3}=\frac{20\;log_{10}\;e}{a_{avg.}-a_{c}}$$

where, $a_{avg}$ is the average amplitude and $a_{c}$ is the threshold magnitude [53,54]. In the context of AE-based damage assessment using the $b_{3}$*-value*, earlier research works were carried out to find the fracture process in rocks [54,55]. Later, some researchers implemented this method in the progressive damage assessment of concrete structures [50].

### 2.3. Machine Learning Based Approaches

Two types of the machine learning techniques, i.e., supervised learning and unsupervised learning, were used in the present research work for the interpretation of AE data. In the case of the supervised learning technique, a model is trained with labeled data to predict future outputs. On the contrary, an unsupervised learning technique does not need labeled data, but it can find the hidden patterns in the input data. There are many supervised as well as unsupervised learning algorithms available; however, the discussion is limited to only those used in the present study, which are presented below.

#### 2.3.1. Supervised Learning Using Support-Vector Machine

**Figure 2:**
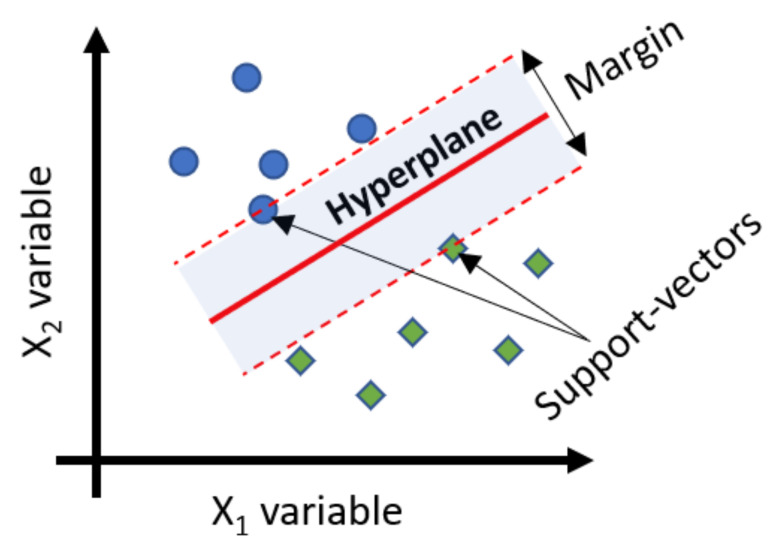
SVM, support vectors, hyperplane (schematic).

Support-vector machine (SVM) is a widely used supervised learning method for classification [56]. The SVM algorithm searches for an optimal separation boundary (usually called the hyperplane or the decision boundary), which maximizes the margin as shown in Figure 2. Such maximization of the margin between the two classes on the training data results in an efficient classification on test data [57]. The points on the margins are called support vectors [57]. In SVM, the training data are first mapped in space and then the test data are mapped in the same space to determine in which class they fall.

The Lagrangian dual problem [58] for linearly and nonlinearly separable classes can be expressed as follows.

■ For linearly separable classes (i.e., linear SVM), minimize
  (7) $$\frac{1}{2}\overset{n}{\underset{i=1}{\sum }}\overset{n}{\underset{k=1}{\sum }}\alpha_{i}\alpha_{k}y_{i}y_{k}x_{i}^{T}x_{k}-\overset{n}{\underset{i=1}{\sum }}\alpha_{i}$$
  with respect to $\alpha_{1},\ldots ,\;\alpha_{n}$ subject to $\sum \alpha_{i}y_{i}=0,\;\alpha_{i}\geq 0\;\forall_{i}$.
■ For nonlinearly separable classes (i.e., nonlinear SVM), minimize
  (8) $$\frac{1}{2}\overset{n}{\underset{i=1}{\sum }}\overset{n}{\underset{k=1}{\sum }}\alpha_{i}\alpha_{k}y_{i}y_{k}\;K\left(x_{i},x_{k}\right)-\overset{n}{\underset{i=1}{\sum }}\alpha_{i}$$
  with respect to $\alpha_{1},\ldots ,\;\alpha_{n}\;$ subject to $\sum \alpha_{i}y_{i}=0,\;0\leq \alpha_{i}\leq C\;\forall_{i}$.

Where ($x_{1}y_{1}$), ($x_{2}y_{2}$), ($x_{3}y_{3}$), …, ($x_{n}y_{n}$), are ‘*n*’ data points with $x_{i}\in {\mathbb{R}}^{P}$ and $y_{i}\in \left\{-1,\;1\right\}$, for all i = 1, …, *n*. The objective remains the same for both linearly and nonlinearly separable classes. However, the condition for coefficients ($\alpha_{i})\;$ changes, where for linearly separable classes $\alpha_{i}\geq 0\;\forall_{i}$; and for nonlinearly separable classes, $0\leq \alpha_{i}\leq C\forall_{i}$, C being a regularization parameter. $K\left(x_{i},x_{k}\right)=\langle \phi \left(x_{i}\right),\phi \left(x_{k}\right)\rangle$, is the dot product (i.e., inner product) of the transformed features using kernel function ϕ. Some well-known kernel functions are the Gaussian radial basis function (RBF), polynomial, sigmoid (i.e., neural network), and linear. The RBF and linear kernel functions were used in the present study and are given as:

(9) $$\mathrm{Gaussian}\;\mathrm{RBF}:\;K\left(x_{i},x_{k}\right)=exp\left(-\frac{\|x_{i}-x_{k}\|{}^{2}}{2\sigma^{2}}\right),\;\sigma \;\mathrm{is}\;\mathrm{kernel}\;\mathrm{width}.$$

(10) $$\mathrm{Linear}:\;K\left(x_{i},x_{k}\right)=x_{i}^{T}x_{k}$$

Nonlinear SVM works in the transformed predictor space to find an optimal hyperplane. The advantage of the RBF kernel is that it has an efficient learning ability in nonlinear cases [59].

#### 2.3.2. Unsupervised Learning Approach

**Figure 3:**
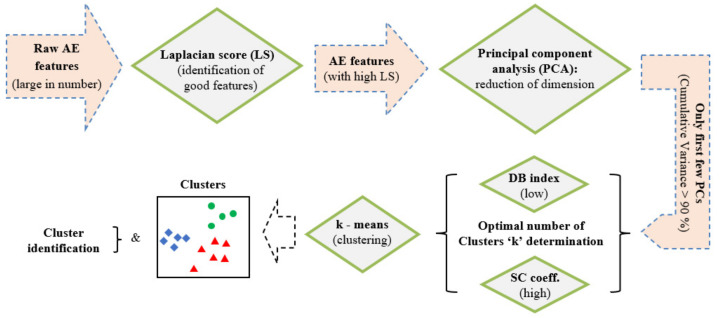
Flow chart showing steps involved in the adopted unsupervised learning scheme.

The unsupervised learning approach adopted in the present study consists of three steps: feature selection, feature optimization (i.e., dimensionality reduction), and clustering. Although clustering of data is the main goal to acquire an insight into the hidden patterns of the data, the other two steps are very important to improve the abovementioned clustering. In the present study, important features were identified using Laplacian score and feature optimization has been carried out using principal component analysis (PCA). Finally, the clustering of AE data was performed with the help of a k-means algorithm considering optimized data (Figure 3).

Laplacian score (LS) is an advanced variance analysis tool that prefers features with larger variance having more locality preserving ability [60]. An important feature is determined with the help of its Laplacian score. A good AE feature that can cluster data has an LS greater than 0.9 [61]. Thus, with the help of LS analysis only the important features can be selected for the subsequent principal component analysis. Principal component analysis (PCA) [39] is a well-known feature optimization technique [41]. It is based on the eigen decomposition of the feature covariance matrix. The principal components are orthogonal to each other, and each principal component is basically a linear combination of the original variables. Principal components have a decreasing trend in variance, i.e., the first principal component has the maximum variance. Projection of each observation on the first principal component axis would therefore result in maximum variance, while projection of the same observations on second principal component axis would yield second largest variance, and so on. Usually, only the first few PC are used for subsequent analysis since the cumulative variance of these PC is quite large. Finally, the k-means algorithm partitions data into k-number of mutually exclusive clusters [62]. k-means minimizes the sum of the distances between the centroid and all member objects of each cluster. The point to be noted here is that the value of *k* of the algorithm is not known initially but chosen depending on some cluster validity indices. The widely used cluster validity indices found in the literature are the Davies–Bouldin (DB) index and the Silhouette coefficient (SC), which are defined below.

■ *Davies-Bouldin (DB) index*: It is defined as the ratio of the sum of within-cluster scatter to between-cluster separation [63].
  (11) $$DB=\frac{1}{k}\overset{k}{\underset{i=1}{\sum }}\begin{array}{c} max\\ j\neq i \end{array}\left\{\frac{d_{i}+d_{j}}{D_{ij}}\right\}$$
  where, k is the number of clusters, and $d_{i}$ and $d_{j}$ are average within-cluster distances of clusters i and j, respectively. $D_{ij}$ is the distance between the centers of the $i^{th}$ and $j^{th}$ clusters. It can be easily understood from this formula (Equation (11)) that a lower value of DB indicates a better separation of the clusters with a good compactness inside the clusters. [64]
■ *Silhouette coefficient (SC):* This is an interpretation and validation method of consistency of data within the clusters. The Silhouette value can be calculated as follows [65]:
  (12) $$S\left(i\right)=\frac{B\left(i\right)-A\left(i\right)}{\max\;\{A\left(i\right),\;B\left(i\right)\}}$$
  where $A\left(i\right)$ is the average dissimilarity of i with all other data within the same cluster. $B\left(i\right)$ is the lowest average dissimilarity of i to any other cluster of which i is not a member. A high Silhouette value indicates that the object is well matched to its own cluster and poorly matched to the neighboring clusters. SC ranges from 0 to 1, and a value of SC greater than 0.6 generally assures that the clustering is of a sufficient quality [66].
  DB and SC are therefore used for obtaining the optimal cluster number, which is simultaneously indicated by a lower Davies–Bouldin index and a higher Silhouette coefficient [67,68].

## 3. AE Monitoring of Mechanical Tests

**Figure 4:**
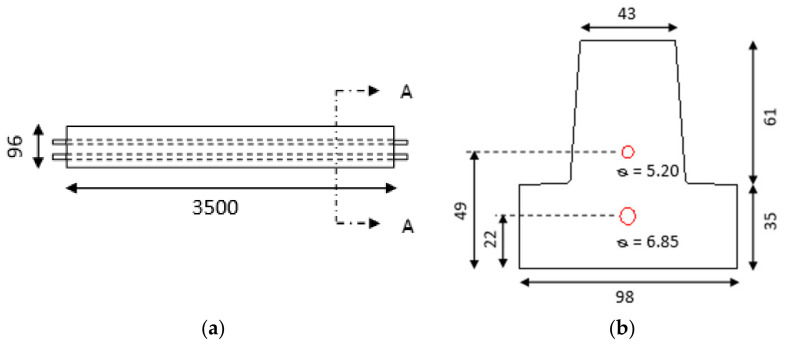
Dimensions of the reinforced concrete T-beam (in mm): (**a**) longitudinal and (**b**) cross sectional dimensions.

**Figure 5:**
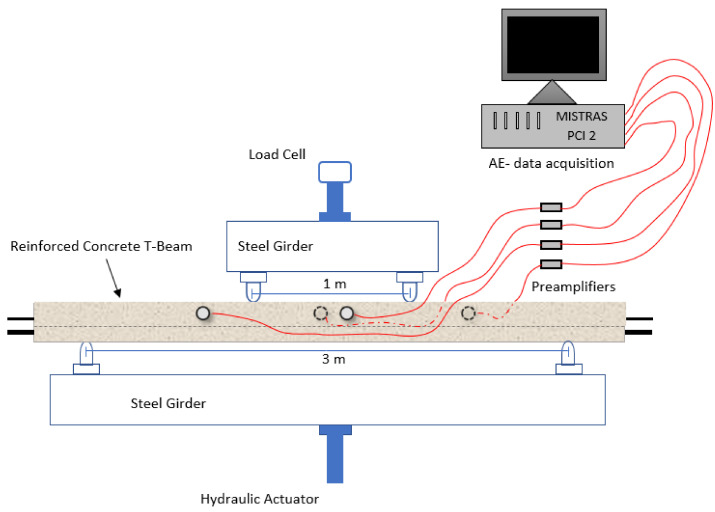
Reinforced concrete T-beam subjected to four-point bending and acoustic monitoring.

**Figure 6:**
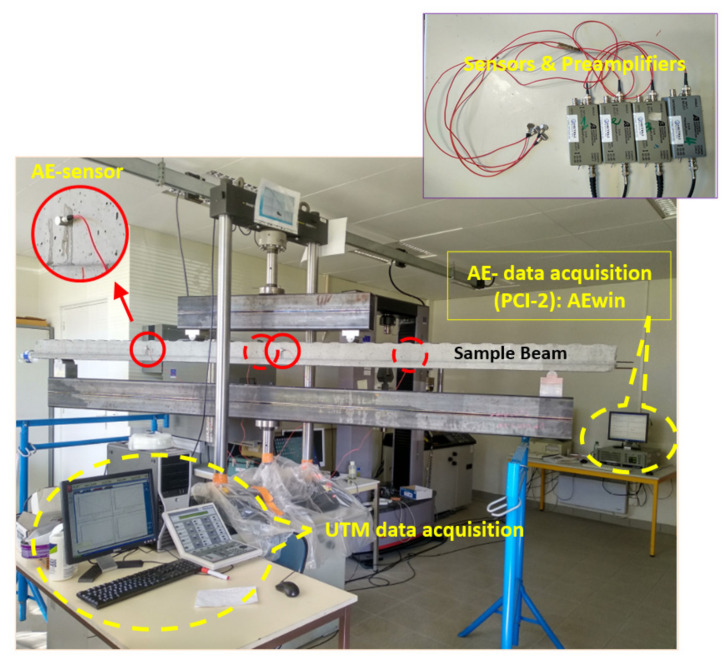
Experimental set-up: UTM, sample beam, and AE setup.

Four-point bending tests were conducted on 3.5 m-long samples of reinforced concrete T-beams with the help of a Universal Testing Machine (INSTRON 8801). The T-beams were manufactured by RECTOR^®^ following the French standard NF EN 15804+A1 and its national complement NF EN 15804/CN. The mechanical properties of the beams are given in Table 1 and the dimensions of the same in Figure 4. The distance between the bottom supports is 3 m and the distance between the loading points is 1 m (Figure 5). The load is applied with the help of a servo-controlled hydraulic actuator of the Universal Testing Machine, in a displacement-controlled manner. Different displacement rates (1 mm/min, 2 mm/min, and 4 mm/min) were applied on identical samples, namely sample 1, sample 2, and sample 3, respectively. The AE set-up (Figure 5 and Figure 6) consists of sensors, preamplifiers, and a data acquisition system (i.e., PCI-2 of Physical Acoustics). Four sensors capture acoustic emission hits created during the occurrence of damage events in the tested sample. The sensors used were broad band-type PAC (MICRO-80) and the selected frequency bandwidth was 20 kHz–1 MHz for all the tests. A threshold of 45 dB and a pre-amplifier gain of 40 dB were assigned as inputs. In order to check the sensitivity and coupling of the sensors, pencil lead break (PLB) was performed before the actual AE monitoring of each sample.

## 4. Results and Discussion

### 4.1. Global Analysis of Damage

**Figure 7:**
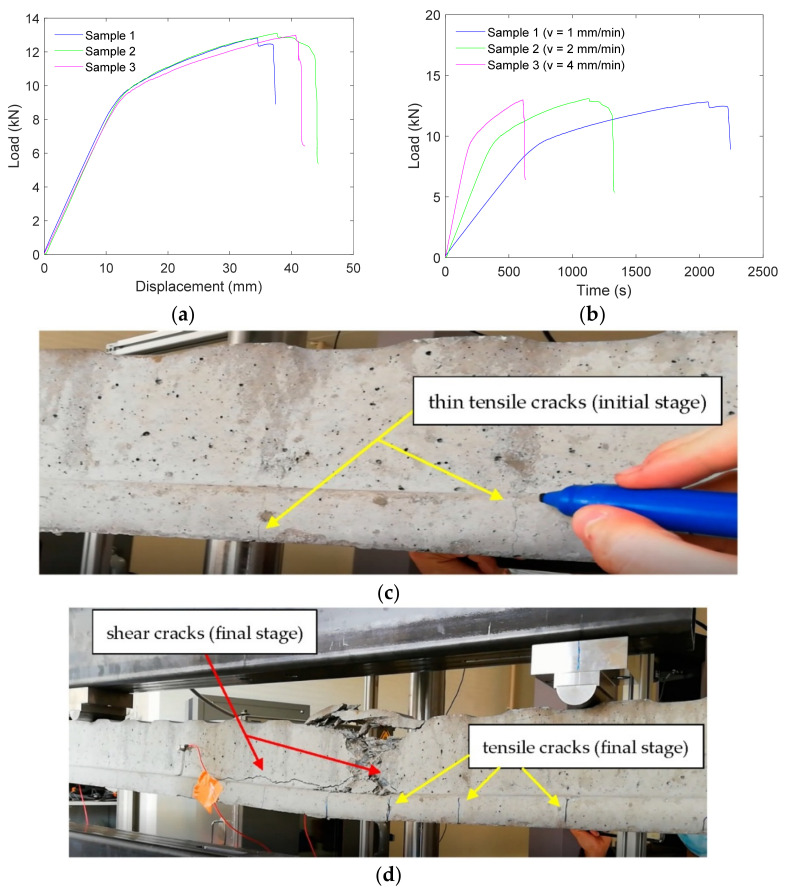
(**a**) Evolution of load as a function of displacement, (**b**) evolution of load as a function time, (**c**) initial cracks, and (**d**) rupture of the beam.

In the present work, the four-point bending test was coupled with AE monitoring, where the main characteristics of the performed mechanical tests are presented in Table 2. In Figure 7a, it can be observed that results corresponding to the three different tests were very close to each other and within the limit of proportionality. It can also be observed that the load-displacement curves of sample 1 and sample 2 had a good agreement even in the major part of the nonlinear zone. Note that the behavior of sample 3 was different from those of samples 1 and 2 due to its relatively high loading rate. It should be noted that, in the load-displacement curve, a sudden drop in the value of load in the nonlinear zone indicates the formation of a major crack in the sample during the test. We also note that the change in the speed ratios between the three different tests has a clear effect on their durations. This can be verified in Figure 7b with the help of the sudden drop in the value of load in the nonlinear zone, where the final cracking happened approximately at 650 s, 1300 s, and 2300 s for tests 1, 2, and 3, respectively. In the literature, it was found that the main crack proceeds with increasing velocity for increasing loading rates [69]. Indeed, the crack velocity increased by orders of magnitude at high loading rates while it propagated in an almost constant way at lower loading rates [70,71].

**Figure 8:**
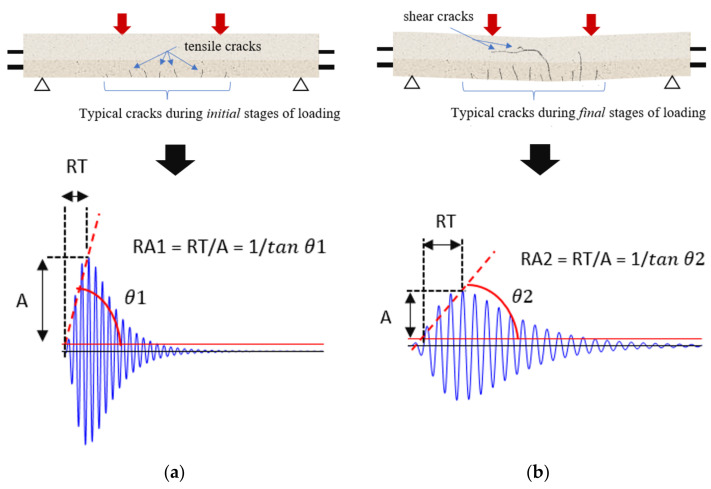
Schematic presentation of cracks and the corresponding dominating AE signals: (**a**) cracks during initial stages of loading and AE signal, and (**b**) cracks during final stages of loading and AE signal.

In order to explain the cracks in terms of their appearance and orientations, and corresponding typical AE-signals, during the successive loading stages, a schematic presentation is given in Figure 8. In the initial stages of loading, as shown in Figure 8a, a few tiny vertical cracks were observed, mostly in the span between the loading points (upper). These are tensile cracks. With the increase in the loading, these tiny cracks increased in size and crack tips propagated upwards. A few more cracks were also seen to develop during this period. When the applied loading reached considerably high values during final stages of loading, shear cracks, mainly oriented horizontal or inclined, as shown in Figure 8b, started to develop and quickly became significantly large, resulting in a significant reduction in strength, indicating impending failure of the sample. The typical AE signals during the initial and final stages of loading are also shown in Figure 8. The AE signals during initial stages of loading were characterized by high frequency and low rise time; on the contrary, the AE signals during the final stages of loading had low frequency and higher rise time. These explain AE signals of the initial and final stages of loading to possess significantly different *RA values*.

**Figure 9:**
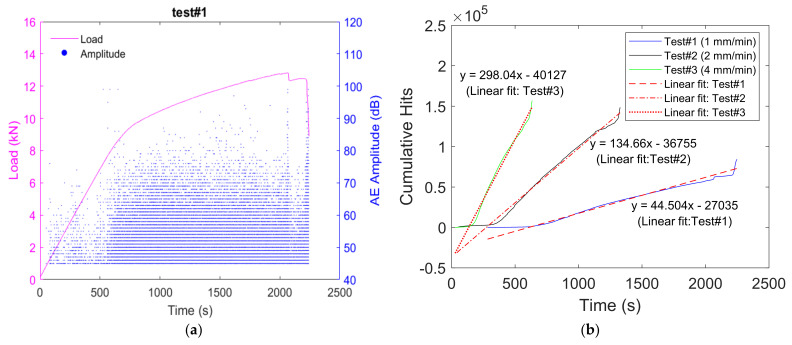
(**a**) Variation of load and AE amplitude with time (this observation is found to be consistent for each of the samples) and (**b**) variation in cumulative AE hits with time and corresponding linear fit.

In our tests, various features (i.e., amplitude, rise time, counts, duration, energy, number of hits, etc.) [72] of the acoustic emission signals can be used for analysis with the aim to monitor the progressive damage of the samples under the four-point bending test. It was observed that the AE activity evolves as function of the applied load, where amplitudes of the involved AE hits become higher in the case of a major crack (Figure 9a). On the other hand, the cumulative number of hits can be considered as a global parameter, which can be sensitive to the mechanical test conditions (i.e., load rate). Figure 9b shows that the evolution of the cumulative hits per unit time is constant for each mechanical test. This is in agreement with the results of the literature, which predict a constant velocity for the cracks for moderate loading rates [70,71]. On the other hand, the evolution of the cumulative hits per unit time was found to be proportional to the loading rate, where the highest value was found for the loading rate corresponding to 4 mm/s, which created the shortest nonlinear zone.

### 4.2. Physical Parameters-Based Analysis of Damage

**Figure 10:**
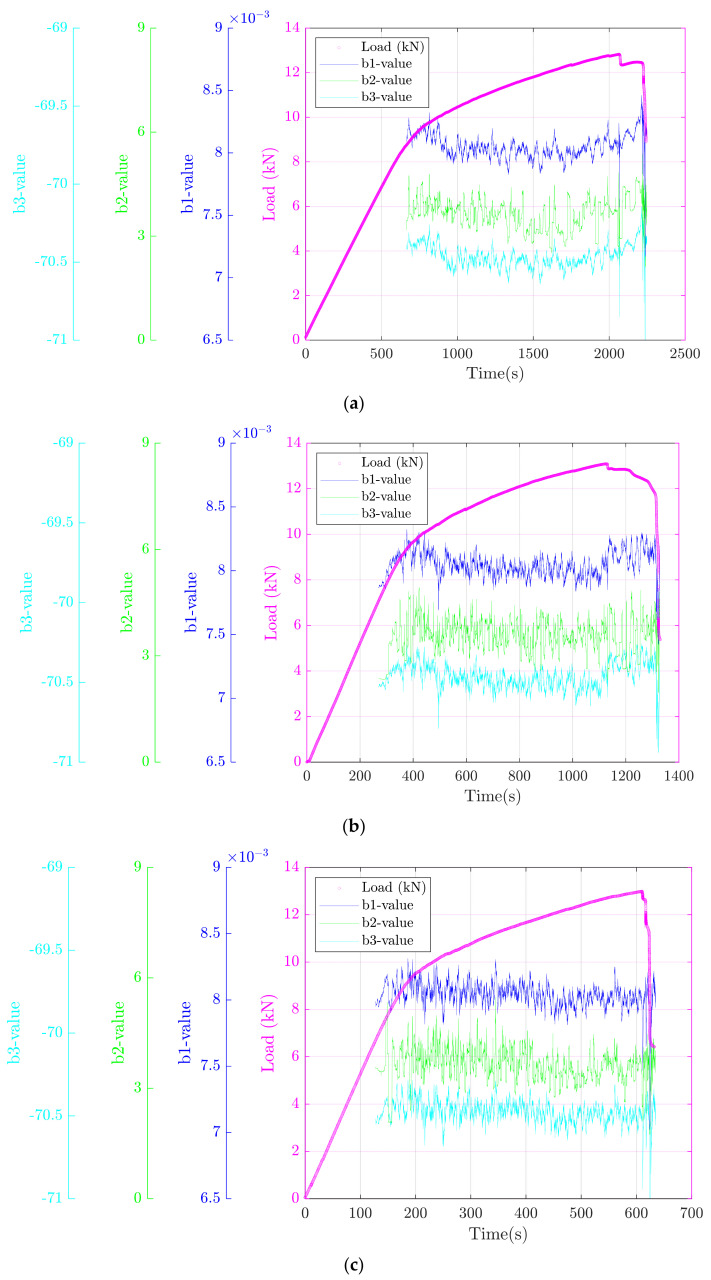
Evolution of the three types of *b-values* during the performed mechanical loading at (**a**) 1 mm/s, (**b**) 2 mm/s, and (**c**) 4 mm/s.

The physical parameters discussed in the theory section can also be used for the interpretation of AE data. Since macro-cracking is accompanied with an important release of stored strain energy within tested materials, relatively small *b-values* can be expected. The prediction of macro-cracking can therefore be performed on the basis of the dominating micro-cracks events having small energies. Large energies are emitted when macro-crack events are created (through the coalescence of micro cracks) which makes the *b-value* drop. Results obtained using the three different *b-value* definitions show that *b*_1_-*value*, *b*_2_-*value*, and *b*_3_*-value* sharply decreased when macro-cracking events occurred (Figure 10). The three definitions were effectively able to identify both small-scale and large-scale events in accordance with previous studies performed on geomaterials [45,73,74]. Finally, we note that a quantitative distribution based on the abovementioned *b-values* can also be performed in order to predict large-scale events and/or ultimate failure, as was carried out to predict the fracture damage of sandstone, where it was found that the ultimate failure happened when the three types of *b-values* were the minimum [45]. This observation is in agreement with our results.

**Figure 11:**
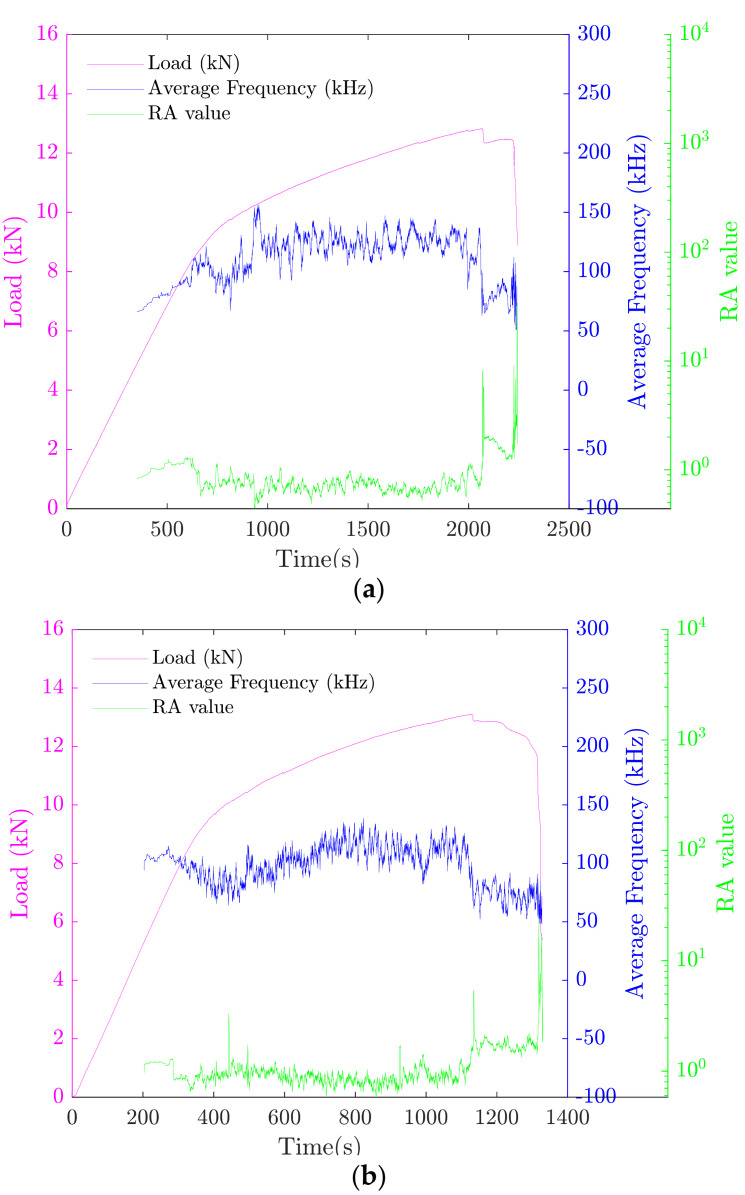

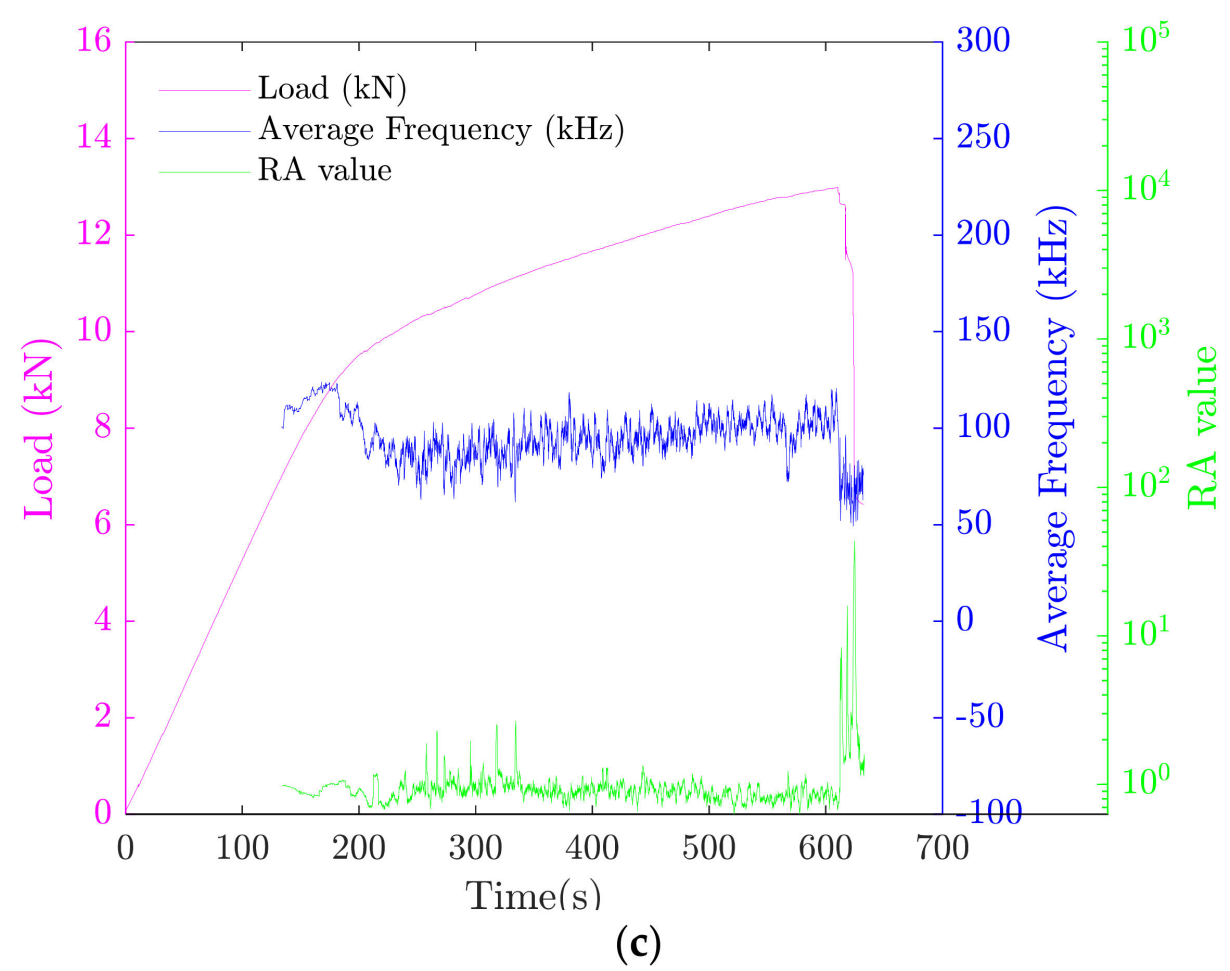
Variation of load, average frequency (AF), and *RA value* with respect to time: (**a**) sample 1, (**b**) sample 2, and (**c**) sample 3.

The monitoring of the fracture mode can be performed by evaluating the average frequency (AF) and *RA value*. Indeed, when the fracture mode is dominated by tension-type cracking, the value of AF is higher, and the *RA value* is lower. However, in the case of a shear-type cracking, there is a sudden decline in AF and a sharp increase in *RA value*. As shown in Figure 11, the pattern was found to be consistent for the different mechanical tests. According to literature, early stages of damage are mainly due to tensile cracking modes. As damage evolves, more shear cracks are created. This observation, which has been confirmed by many studies [32,75], justify the results presented in Figure 11, in which high sensitivity of both parameters to the type of cracking can be observed.

### 4.3. Machine Learning Based Approaches

In order to classify the AE data, machine learning methods (i.e., supervised and unsupervised) were employed. In the case of supervised learning, support-vector machine (SVM) was used to classify the AE data. On the other hand, in the case of unsupervised learning, principal component analysis (PCA) and k-means were implemented for dimensionality reduction and clustering of the AE data, respectively. However, a point to be noted here is that a large number of features were used in the unsupervised machine learning approach, whereas the supervised machine learning was only based on two features, namely average frequency and *RA value*.

#### 4.3.1. Supervised Learning Using SVM

**Figure 12:**
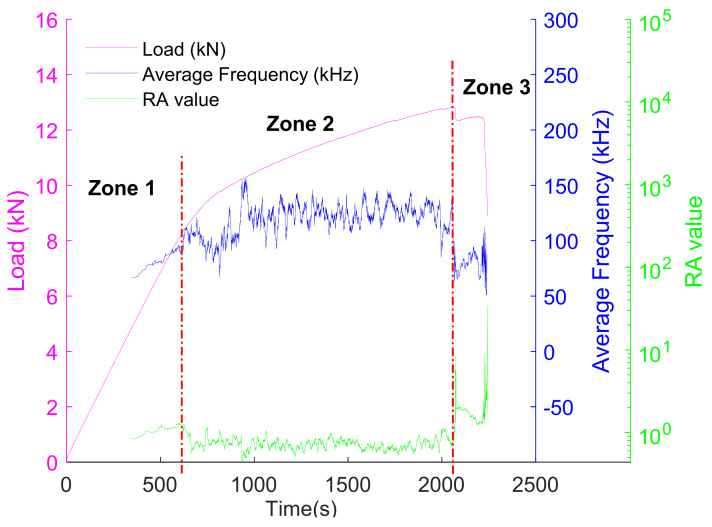
Labeling of the data set into three separate classes (i.e., Zone 1, Zone 2, and Zone 3).

Support-vector machine (SVM) is vastly used in supervised learning. However, to implement this method, a labeled data set is required for training the model. Therefore, at first, the AE data set consisting of average frequency (AF) and *RA value*, which was used in this supervised learning, were labeled. This step is mandatory for the algorithm to work. It is also very important since the performance of the algorithm depends on this. Since the load-displacement characteristic of the tested beam samples has three separate zones (i.e., linear, nonlinear, and shear), the labeling was carried out based on that. In other words, AE data which correspond to linear, nonlinear, and shear zones were labeled as Zone 1, Zone 2, and Zone 3, respectively (Figure 12).

**Figure 13:**
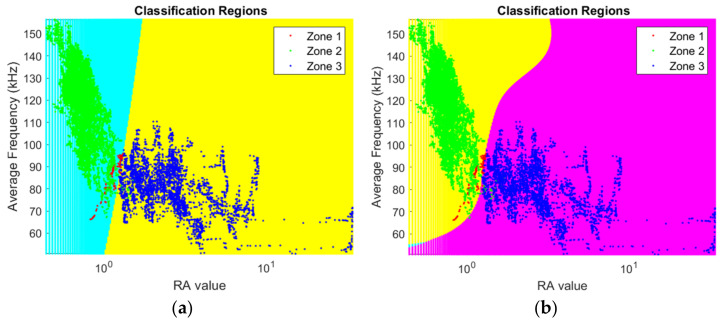
Classification using SVM (for sample 1 data): (**a**) using linear kernel and (**b**) using Gaussian kernel.

The results obtained using SVM showed that Zone 1 could not be well separated from Zone 2 (Figure 13). This is reasonable because Zone 1 data corresponds to tensile micro cracks, while Zone 2 data corresponds to micro as well as moderate cracks. Basically, the characteristics of Zone 1 data are similar to the characteristics of a fraction of the Zone 2 data. Hence, in the subsequent analysis, using SVM only two labels were used. In other words, the linear and nonlinear part have the same label, namely Zone 1 + Zone 2, and the shear part was assigned the same label as before, i.e., Zone 3. On the other hand, it was also observed that the performance of SVM is better for the Gaussian kernel compared with linear kernel. Therefore, the SVM analyses were performed using the Gaussian kernel only. Very few studies are available on the application of SVM for the classification of AE data obtained from concrete structures. SVM was adopted in refs. [35,36] to classify cracks in reinforced concrete using RA–AF data. AE data were classified into two classes, i.e., tensile and shear, in the RA–AF plane using a linear kernel. A point to be mentioned is that the choice of the kernel type depends on the pattern in the data.

**Figure 14:**
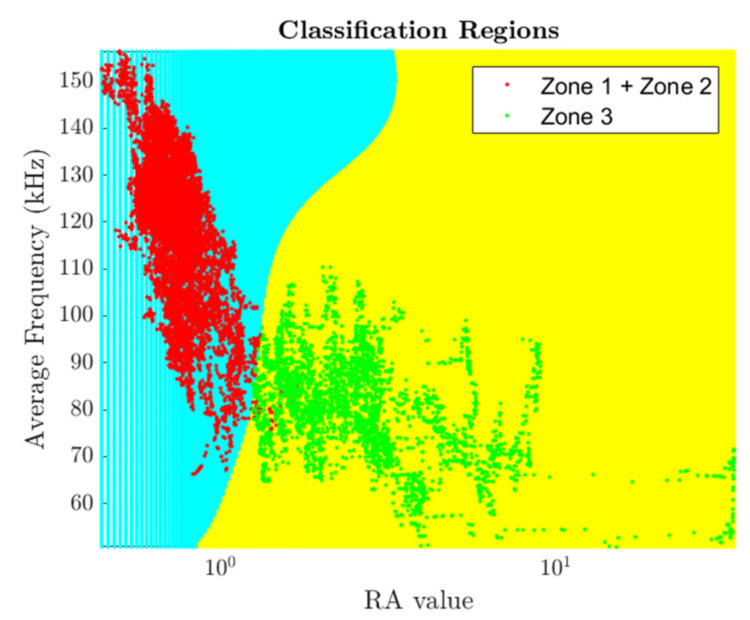
Two labels of SVM classification using Gaussian kernel in the case of sample 1.

The Gaussian kernel trick of SVM was found to be very efficient in the classification of AE data (i.e., average frequency and *RA value*) with the help of its nonlinear hyper plane (Figure 14). Although the supervised machine learning approach using SVM worked well for the classification of AE data, it is difficult to classify data if multiple mechanisms are present in a particular zone. For example, in the nonlinear zone (i.e., Zone 2), where multiple damage mechanisms are present, it remains difficult to label data of Zone 2 with different labels that correspond to different damage mechanisms. Hence, there is a need for an unsupervised learning approach because it does not require pre-assigned labeled data for the classification.

#### 4.3.2. Unsupervised Machine Learning for Clustering of AE Data

A large number of features can be considered for the unsupervised approach as shown in Table 3. The determination of the Laplacian score (LS) corresponding to each feature helps to identify the most relevant ones. The latter are characterized by a large variance and have more representative power. In this study, the features which have LS> 0.9 were chosen for subsequent principal component analysis (PCA) to reduce the dimensionality of data. Finally, only the first few principal components, which have cumulative variance >90%, were used for the subsequent k-means method to cluster AE data. A point to be noted here is that an optimal number of clusters for the k-means was determined with the help of the Davies–Bouldin (DB) index and Silhouette Coefficient (SC), as discussed in the theory section.

**Figure 15:**
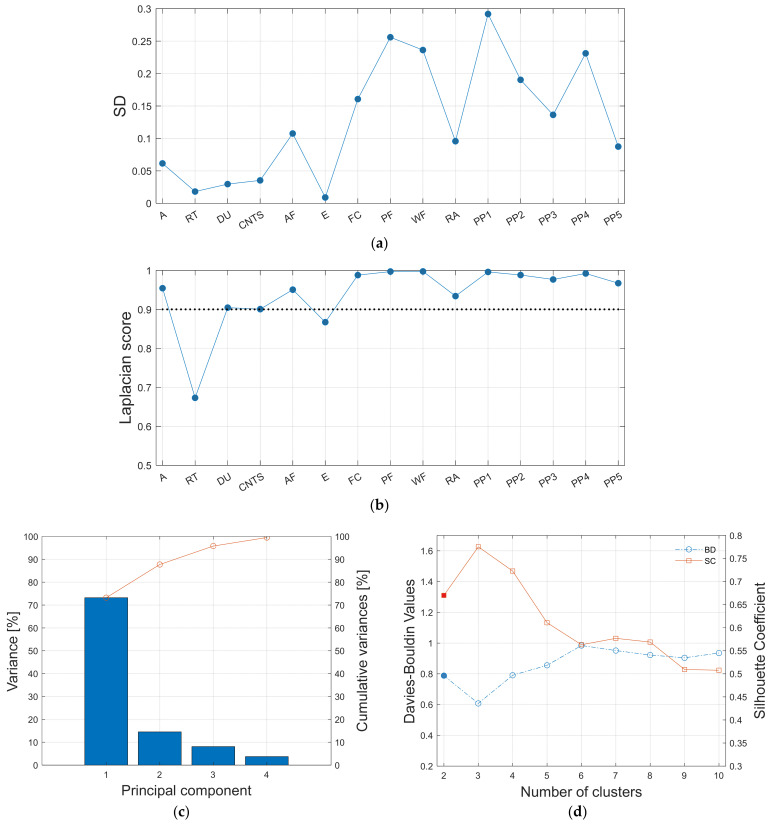
Different steps of the adopted unsupervised learning method (shown for AE data of sample 1): (**a**) Standard deviation of features, (**b**) feature selection using Laplacian scores, (**c**) screen plot obtained from PCA analysis, and (**d**) selection of the optimal number of clusters for k-means using cluster validity indices, i.e., Davies–Bouldin (DB) index and Silhouette Coefficient (SC).

Typical results of the pre-requisite steps before clustering by k-means are given in Figure 15 using the AE data of sample 1. Although the Laplacian score of each feature was used for the selection of features that have larger variance with more locality preserving ability, the standard deviation of each feature is also given in Figure 15a to show the correspondence between Laplacian score (LS) and standard deviation (SD) of each feature. A similar trend can be observed through both LS and SD data. Based on the criterion LS > 0.9, as given in Figure 15b, it can be observed that the three first principal components represent more than 90% of the total variance, as shown in Figure 15c. Hence, only these first three principal components were considered for the subsequent k-means algorithm. The optimal number of clusters for k-means analysis was determined based on the Davies–Bouldin (DB) index and Silhouette coefficient (SC) (Figure 15d), which should be simultaneously the minimum and maximum, respectively. Finally, note that similar trends were also found for the AE data of sample 2 and sample 3, but are not included here.

**Figure 16:**
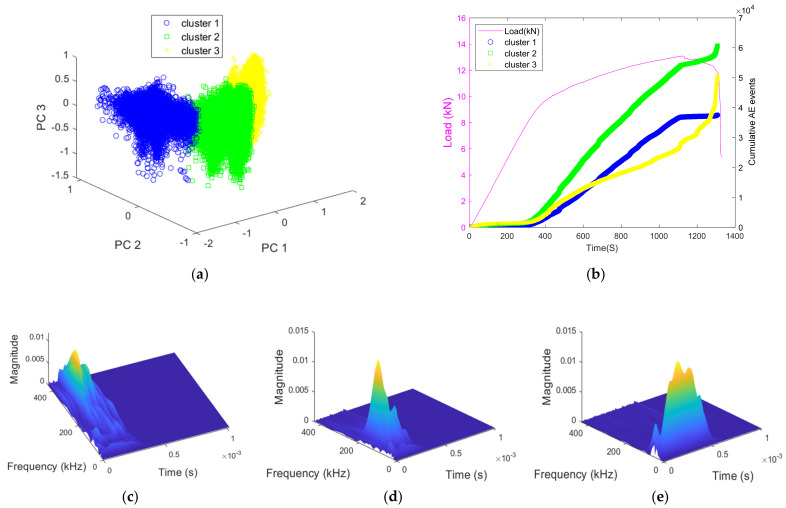
Clustering using k-means and significance of the obtained clusters (shown for AE data of sample 2): (**a**) obtained clusters, (**b**) variation in cumulative hits of the clusters; a typical wavelet transformed signal of (**c**) cluster 1, (**d**) cluster 2, and (**e**) cluster 3.

According to the explanations given above, the results obtained using k-means for three clusters are presented in Figure 16a. The three clusters do not have the same characteristics in terms of time and frequency parameters. Unlike cluster 3, cluster 1 has the highest frequency components, as shown in Figure 16c,e. On the other hand, cluster 2 has frequency components of intermediate values, as shown in Figure 16d. Figure 16b shows that the cumulative hits for cluster 1 increased consistently until the major shear crack happened before keeping a constant value. The cumulative hits for cluster 2 were found to be gradually increasing from start till the occurrence of the major crack, where a moderate jump could be observed during the major shear crack. The cumulative hits for cluster 3 increased slowly from the start and a significant jump could be observed during the occurrence of the major shear crack. Finally, note that these observations were found to be consistent for all three samples.

The three clusters mentioned earlier can be associated with microcracking at the matrix–aggregate interface/reinforcement and/or micro-cracking in the matrix. An amplitude-based correspondence between the amplitudes of the signals and the mechanisms can be performed as in ref. [76]. However, differences between ranges of amplitude of classes are to be expected depending on the specimens’ geometries and recording system parameters [77,78,79,80]. On the other hand, a tensile crack is created at the initial loading stage and evolves throughout the loading, whereas shear movements are restrained by the high-strength steel reinforcements inside the beam [33]. However, we should note that due to shear displacement, more friction at shear cracking is to be expected, where the resulting shear waves filter out high-frequency components more easily than the longitudinal waves created by the tensile cracking [81].

In light of these observations, the results obtained suggested that cluster 3, which has the weakest frequency components (around ~40 kHz) is related to friction while cluster 1 is basically related to tensile cracking [82]. Cluster 2 with its intermediate frequency components (around ~170 kHz) appears as a representation combining both shear and tensile cracking. This has been reported in the literature as a tensile cracking mixed with friction between crack faces [83]. Therefore, the performed analysis completes the one based on the evolution of *RA value* and average frequency. Indeed, the RA parameter may be insufficient to distinguish between tensile cracking and friction due to the attenuation of elastic waves emitted by the creation and propagation of cracks within heterogeneous materials such as concrete [84]. Due to attenuation, an increase in *RA value* with decreasing amplitude (i.e., decrease in the average frequency) will then lead to erroneous signal labeling, which makes the effectiveness of such a classification questionable beyond a certain propagation distance [82].

## 5. Conclusions

Acoustic emission (AE)-based techniques were adopted to monitor progressive damage mechanisms in reinforced concrete T-beams subjected to four-point bending test. The test was conducted on three identical samples but with different displacement rates to observe the effect on global damage behavior. Various analysis schemes were adopted. Based on the analysis of the results, the following conclusions are drawn:

- The load-displacement curves obtained for the three samples were similar, hence repeatable. In the load-time curves, it was found that rate of displacement has a direct impact on the rate of acoustic emission events; a higher displacement rate results in a higher rate of AE events.
- Various physical parameter-based algorithms were adopted to discern different damage mechanisms in reinforced concrete T-beams under test. Although different algorithms may use different AE feature/s, they are able to provide useful information about the progressive damage stages. For instance, AE features used in average frequency (AF) and *RA value* are different; however, these two algorithms separately able to distinguish the stages of tension-type cracking and shear-type cracking, effectively.
- In the case of *b*_1_-*value*, *b*_2_*-value*, and *b*_3_-*value* analysis, each of these algorithms use AE amplitude. It was found that all these *b-value* algorithms can identify micro-damage and macro-damage cases by showing a sudden drop in their respective indices in case of a macro-damage. However, *b*_1_-*value* and *b*_3_-*value* were found to be more sensitive in discriminating micro-damage and macro-damage compared with *b*_2_-*value*.
- With a view to classify and cluster AE data, supervised and unsupervised machine learning methods were adopted, respectively. In case of the supervised machine learning using SVM, two classes were successfully made considering only the average frequency (AF) and *RA value* as features in the algorithm. The Gaussian kernel trick of SVM was found to be very efficient in the classification of AE data with the help of its nonlinear hyper plane. Although the supervised machine learning approach using SVM worked well for the classification of AE data, it is difficult to classify the data if multiple mechanisms are present in a particular zone of load-displacement curve, because the labeling of data becomes very difficult in such a scenario. Hence, there is a need of an unsupervised learning approach as it does not require labeled data for the classification.
- In the case of the unsupervised machine learning approach, a large number of features were considered. With a view to reduce the dimension and optimize the AE data, Laplacian score and principal component analysis (PCA) were performed. Finally, the k-means algorithm was used for clustering of data, considering only the first few PCs. Three clusters were obtained. Based on the cumulative hits of different clusters and frequency domain analysis of randomly chosen signals from each of the clusters, it can be concluded that the three clusters correspond to three different types of damage cases, namely, tensile cracking (cluster 1), both shear and tensile cracking (cluster 2), and friction (cluster 3).

Future studies can be focused on analysis of new time/frequency parameters that are to be defined in accordance with the involved micro-mechanisms (e.g., shear and/or tensile) in order to refine the damage analysis in reinforced concrete.

## Figures and Tables

**Table 1:** Mechanical properties of the reinforced concrete T-beams as provided by the manufacturer (i.e., RECTOR^®^).

Ingredients	Characteristics
Concrete	Compressive strength = 50 MPa
Steel	Ultimate tensile strength = 525 MPa Yield strength in tension = 500 MPa

**Table 2:** Mechanical test results.

Samples	Rate of Displacement (mm/min)	Time (s)	Load at Limit of Proportionality (N)	Displacement at Limit of Proportionality (mm)	Maximum Load (N)	Maximum Displacement (mm)
S1	1	2242	8151	10.06	12,830	37.10
S2	2	1329	8402	10.79	13,094	43.74
S3	4	632	8299	10.80	12,992	41.12
Average			8284	10.55	12,972	40.65

**Table 3:** Features selected for the unsupervised learning [72].

Feature	Unit	Feature	Unit	Feature	Unit
Amplitude (A)	dB	Average Frequency (AF)	kHz	Partial Power 1 (PP1)	-
Rise time (RT)	µs	Frequency Centroid (FC)	kHz	Partial Power 2 (PP2)	-
Duration (DU)	µs	Peak Frequency (PF)	kHz	Partial Power 3 (PP3)	-
Energy (E)	aJ	Weighted Frequency (WF)	kHz	Partial Power 4 (PP4)	-
Counts (CNTS)	-	*RA value* (RA)	µs/V	Partial Power 5 (PP5)	-

## Data Availability

Not applicable.
